# Overcoming NOAC-resistant left atrial appendage thrombus in atrial fibrillation: clinical gaps and therapeutic insights

**DOI:** 10.3389/fcvm.2025.1664386

**Published:** 2025-09-26

**Authors:** Klaudia Vivien Nagy, Bence Arnóth, Ferenc Komlósi, László Gellér, Béla Merkely

**Affiliations:** Heart and Vascular Center, Semmelweis University, Budapest, Hungary

**Keywords:** atrial fibrillation, left atrial appendage thrombus, oral anticoagulation, NOAC resistance, VKA therapy, antithrombotic strategy

## Abstract

Atrial fibrillation (AF) is the most common supraventricular arrhythmia and a major cause of stroke and systemic embolism. The left arial appendage (LAA) is the predominant site of thrombus formation in AF. According to current guidelines, oral anticoagulant (OAC) therapy is recommended in patients with elevated thromboembolic risk. Non-vitamin K antagonist oral anticoagulants (NOACs) are currently preferred over Vitamin K antagonists (VKAs) due to favorable safety profiles. Nevertheless, a subset of patients develop LAA thrombi despite optimal anticoagulant therapy, a clinical scenario not addressed by current guidelines. Recent retrospective studies, including our own cohort analysis, suggest, that modifying antithrombotic regimen may improve thrombus resolution in these cases. However, no prospective trials have yet defined the optimal strategy. This mini-review summarizes the available evidence, highlights the limitations of current practice, and proposes directions for future research in this underrecognized clinical dilemma.

## Introduction

Atrial fibrillation (AF) is the most prevalent sustained supraventricular arrhythmia worldwide and is strongly associated with an increased risk of stroke and systemic thromboembolism (1). In non-valvular AF, More than 90% of thrombi originate from the left atrial appendage (LAA), making it the principal source of embolic events (2). The presence of LAA thrombus (LAAT) is an independent predictor of stroke and systemic thromboembolism and constitutes a contraindication to rhythm control strategies, including electrical cardioversion and catheter ablation (3). Current international guidelines recommend oral anticoagulation (OAC) therapy for AF patients their CHA_2_DS_2_-VA clinical stroke risk score (4).

## Literature review

### Prevalence of LAA thrombus and limitations of current therapy

Although NOAC therapy effectively reduces stroke risk in AF, LAAT can still occur in a subset of patients. NOAC resistant LAAT is defined as thrombus persistence despite ≥3 weeks of appropriately dosed anticoagulation, adjusted for age, weight, and renal function.

A large meta-analysis of 14,653 AF patients revealed, that approximately 3% had persistent LAAT despite optimal OAC therapy (5). There was no difference observed in LA thrombus prevalence between VKA- and NOAC treated patients. Patients with non-paroxysmal AF had a 4-fold higher thrombus prevalence (OR = 4,81) compared to patients with paroxysmal AF. These cases represent a distinct clinical subset, yet current guidelines provide no specific recommendations for their management. In practice, treatment decisions are often empirical and based on physician experience rather than evidence.

### Diagnosis of LAA thrombus

Transesophageal echocardiography (TEE) remains the gold standard for LAAT detection (6), offering high sensitivity and specificity without the use of ionizing radiation or iodinated contrast agents. Real-time Doppler facilitates the measurement of flow velocities and differentiation between other LAA findings, such as spontaneous echo contrast (SEC) and sludge. The principal limitation of TEE is the need for esophageal intubation, which often necessitates sedation to minimize patient discomfort.

Alternative imaging modalities, including intracardiac echocardiography (ICE) and cardiac computed tomography (CT) are being increasingly utilized. Cardiac CT angiography (CTA) is a widely used, noninvasive modality to exclude LAAT and can also provide pre-ablation anatomical mapping. However, it carries a non-negligible risk of radiation exposure and contrast-induced adverse events. Furthermore, the positive predictive value of CTA is considered variable (7). To improve specificity, a delayed scanning protocol was implemented to reduce false-positive results related to slow flow (8, 9).

ICE is an increasingly used intra-procedural imaging modality, especially in the context of left atrial ablations. While primarily employed to enhance procedural safety and guide transseptal puncture, ICE also demonstrates high sensitivity for ruling out LAAT (10), potentially obviating the need for pre-procedural TEE. However, image quality and diagnostic accuracy are highly operator dependent, and suboptimal catheter positioning may result in missed thrombi (11). Moreover, ICE catheters are single-use and associated with substantial cost (12). Although widely used in EP practice, ICE is not considered as a first-line diagnostic tool for LAAT detection.

Imaging is recommended prior to electrical cardioversion (ECV) or catheter ablation (CA) in patients who have not completed at least three weeks of effective OAC therapy. While the incidence of procedure-related cerebrovascular events is relatively low (13, 14), most EP centers perform routine LAA imaging—even in adequatly anticoagulated patients—to minimize residual thromboembolic risk (15).

Current guidelines permit ECV without TEE in patients who have been on adequate anticoagulation for at least three weeks (4). This recommendation is supported by data from the ENSURE-AF trial, which demonstrated similar stroke rates with and without pre-procedural TEE in adequately anticoagulated patients (16). However, it should be noted that this trial was not specifically designed to assess the prevalence of LAAT or to evaluate the role of pre-procedural LAA imaging in high-risk individuals with a history of thromboembolic events or patients with elevated CHA_2_DS_2_-VASc scores.

### Management strategies of an LAA thrombus

Both VKA and NOAC therapy have demonstrated efficacy in resolving LAAT. High rates of thrombus resolution were achieved using prolonged VKA therapy with a target INR of 2–3 (17), or standard-dose edoxaban therapy applied for 12 weeks (18). However, these studies excluded patients on previous OAC therapy. In a more recent retrospective analysis, Kolakowski et al. assessed AF patients with LAAT despite OAC therapy (19). They collected data from 129 patients across 181 treatment cycles. Management strategies were classified into four categories: switching to an anticoagulant with a different mechanism of action, switching within the same class, initiating combination therapy, or continuing the current treatment regimen without any changes. They found that any modification of the baseline OAC treatment significantly improved LAAT resolution compared to no change in treatment. However, no specific modification strategy proved superior. Importantly, only 74 patients (57.4%) in this cohort had previously received NOAC therapy, while the remaining were on VKA.

**Figure 1:**
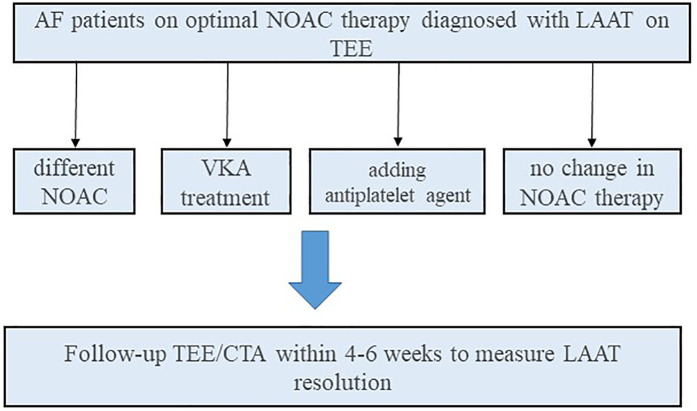
Flowchart demonstrating the diagnostic and therapeutic pathway of AF patients with NOAC-resistant LAA thrombus. CTA, CT angiography; LAAT, left atrial appendage thrombus; NOAC, non-vitamin K oral anticoagulants; OAC, oral anticoagulants; VKA, vitamin K antagonists; TEE, transesophageal echochardiography.

Our research group conducted a retrospective observational study, which provides further insight into this issue by focusing exclusively on AF patients with LAAT despite optimal NOAC use (20). Among 9,547 patients screened, we identified 536 with solid LAAT. Of these, 179 met inclusion criteria, and follow-up imaging was available in 120 cases (Figure 1). The median patient age was 69 (62–74) years. All patients had received NOAC therapy for at least 3 weeks, with doses adjusted based on age, weight and renal function according to the current guidelines.

**Figure 2:**
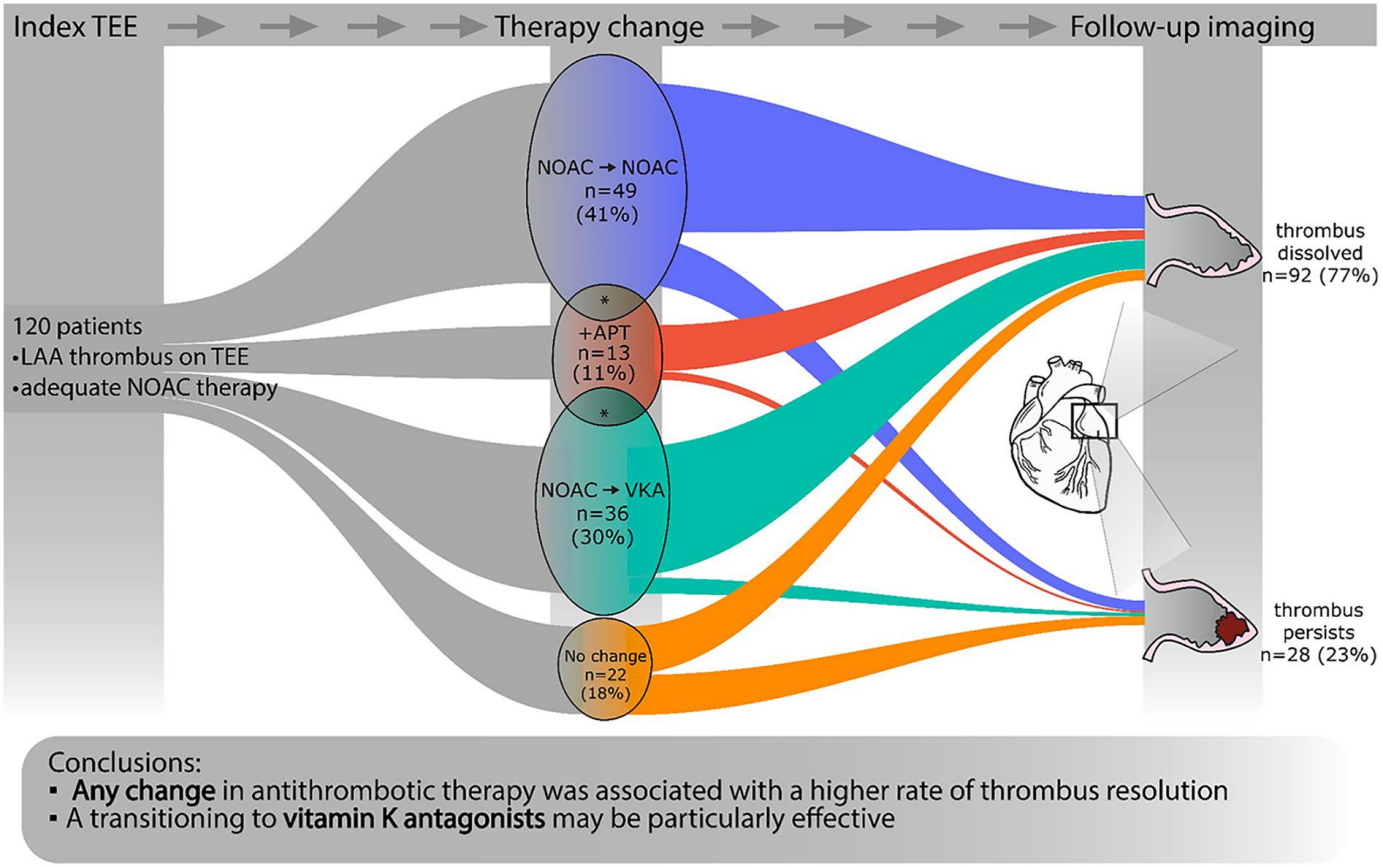
Diagram demonstrating the phases of the study. At a given time point, the width of the colored bands is proportional to the number of patients in the respective subcohort. Asterisks (*) denote the overlap between groups. Those cases where both a change in anticoagulant and augmentation with antiplatelet therapy were applied are displayed according to the change in anticoagulant. APT, anti-platelet therapy; NOAC, novel oral anticoagulant; TEE, transesophageal echocardiography; VKA, vitamin K antagonist.

Management strategies included switching to another NOAC, transitioning to a VKA, initiating antiplatelet therapy, or continuing the current anticoagulant regimen (Figure 2). Our endpoint was successful LAAT resolution, confirmed by the follow-up imaging. This was either a repeat TEE or a left atrial CT angiography at least 3 weeks after the index imaging. Following the index TEE, antithrombotic therapy was modified in 98 (82%) cases. A switch to a different NOAC occurred in 49 (41%) patients, most commonly to dabigatran (69%), followed by apixaban (24%), rivaroxaban (6%), and edoxaban (2%). Eight (7%) of these patients also received adjunctive antiplatelet therapy. Transition to VKA occurred in 36 (30%) cases, three (2%) of whom also received adjunctive antiplatelet therapy. Original treatment was augmented with antiplatelet therapy alone in 13 (11%) patients. Twenty-two patients (18%), remained on their original NOAC therapy, which included apixaban (41%), rivaroxaban (32%), dabigatran (18%), and edoxaban (9%). No patients underwent LAA occlusion.

Follow-up imaging, conducted at a median of 65 (44–95) days after the LAAT diagnosis, consisted of TEE (*n* = 110, 92%) or CT angiography (*n* = 10, 8%). LAAT resolution was observed in 92 patients (77%). Persistent thrombus was present in 28 (23%) cases. Importantly, a significant association was observed between the modification of anticoagulant therapy and LAAT resolution. This suggests that adapting the anticoagulation strategy may offer clinical advantage over maintaining the baseline regimen. Additionally, switching to VKA therapy appeared to be a reasonable approach in selected patients.

Real-world management strategies—albeit in the absence of clear recommendations—align well with the current body of evidence. According to a recently published EHRA survey where clinicians were asked about therapeutic changes in case of NOAC-resistant LAA thrombus, the most common strategies included switching to a different NOAC or transitioning to VKA (21). In addition, LAA closure was also considered as an option in these patients as an off-label therapy. In patients undergoing cardiac surgery for other indications, LAA excision may offer a potential treatment alternative for persistent LAAT. These results reflect the absence of formal guidance and a variability in current therapeutic approaches.

## Discussion

The presence of LAAT in patients with AF presents a significant therapeutic challenge, particularly when it occurs despite adherence to guideline-recommended NOAC therapy. In such cases, rhythm control strategies must be deferred, and clinicians are left with limited evidence to guide the next steps.

Recent studies increasingly support rhythm control as the preferred therapeutic strategy in AF management due to its long-term clinical benefits (22, 23). However, the presence of a LAAT restricts treatment options to rate control alone.

LAATs resistant to OAC therapy are not uncommon and are described both under VKA and NOAC therapy (17, 18) in a large meta-analysis. In this study, non-paroxysmal AF and a CHA_2_DS_2_-VASc score ≥3 were associated with a higher prevalence of LAAT among anticoagulated AF patients (5). Furthermore, patients undergoing ECV were more likely to present with LAATcompared with those undergoing catheter ablation. In another observational study, Angelini et al. identified CHA_2_DS_2_-VASc score ≥3 and obesity as independent predictors of LAAT resistant to optimal anticoagulant therapy (24). Nonetheless, the exact mechanism of thrombus formation despite effective anticoagulation remains elusive.

Both VKA and NOAC therapy have demonstrated efficacy in resolving LAAT (17, 25). However, most previous studies focused on OAC-naive patients, excluding patients already on optimal NOAC therapy at the time of the index TEE.

As mentioned above, emerging data from observational studies suggest that any modification of anticoagulant therapy may offer clinical benefit in this context Kolakowski et al. reported that any change in OAC therapy—regardless of strategy—resulted in thrombus resolution (19). Consistent with these findings, our study focusing exclusively on NOAC-resistant LAAT also demonstrated higher thrombus resolution rates with treatment modification, with transition to VKA proving most effective in our cohort (20).

While still lacking clear recommendations for NOAC-resistant LAATs, the latest guidelines of the European Society of Cardiology addresses the question of stroke despite optimal NOAC therapy. In this population, the authors discourage the change to a different NOAC or the augmentation with antiplatelets to prevent recurrent stroke. This appears to contradict the results of the observational studies of patients with LAATs, including our recent findings. Nevertheless, directly assessing for an LAAT may offer a more sensitive method for evaluating the effectiveness of the chosen antithrombotic strategy. Larger studies powered for thromboembolic outcomes could provide a definite answer to this questions.

## Conclusion

LAA thrombus remains a strong independent predictor of thromboembolic events in AF. Although oral anticoagulant therapy is safe and effective for both stroke prevention and LAA thrombus resolution, thrombus formation may still occur despite adequate anticoagulation. In such cases, clinical decision-making is particularly challenging, as no clear evidence-based guidelines exist for the management of NOAC-resistant LAAT.

Observational data suggest that persistent LAAT under adequate NOAC therapy should prompt re-evaluation of anticoagulation, with consideration of therapy modification. Prospective studies are needed to confirm these observations and to inform evidence-based recommendations. Ultimately, improving the management of this high-risk subgroup may enable safer implementation of rhythm control strategies in AF.
